# Sulfur-Doped Carbon Nanohorn Bifunctional Electrocatalyst for Water Splitting

**DOI:** 10.3390/nano10122416

**Published:** 2020-12-03

**Authors:** Antonia Kagkoura, Raul Arenal, Nikos Tagmatarchis

**Affiliations:** 1 Theoretical and Physical Chemistry Institute, National Hellenic Research Foundation, 48 Vassileos Constantinou Avenue, 11635 Athens, Greece; 2 Laboratorio de Microscopias Avanzadas (LMA), Universidad de Zaragoza, Mariano Esquillor s/n, 50018 Zaragoza, Spain; arenal@unizar.es; 3 Instituto de Nanociencia y Materiales de Aragon (INMA), CSIC-U, de Zaragoza, Calle Pedro Cerbuna 12, 50009 Zaragoza, Spain; 4 ARAID Foundation, 50018 Zaragoza, Spain

**Keywords:** carbon nanohorns, sulfur-doping, electrocatalysis, water-splitting, oxygen evolution reaction, hydrogen evolution reaction

## Abstract

Sulfur-doped carbon nanohorns (S-CNHs) were prepared by an easy one-pot solvothermal process and were employed as efficient electrocatalysts towards water splitting. Initially, oxidation of CNHs followed by thermal treatment with the Lawesson’s reagent resulted in the formation of S-CNHs with the sulfur content determined as high as 3%. The S-CNHs were thoroughly characterized by spectroscopic, thermal and electron microscopy imaging means and then electrocatalytically screened. Specifically, S-CNHs showed excellent activity and durability for both O_2_ and H_2_ evolution reactions, by showing low overpotential at 1.63 and −0.2 V vs. RHE for oxygen and hydrogen evolution reaction, respectively. Additionally, S-CNHs showed significantly lower Tafel slope value and lower current resistance compared to oxidized and pristine CNHs for both electrocatalytic reactions. The outstanding electrocatalytic properties and high conductivity, along with the high S-doping level, render S-CNHs a promising bifunctional electrocatalyst for water splitting.

## 1. Introduction

Electrochemical water splitting is a cost-effective clean method for breaking down water to hydrogen (H_2_) and oxygen (O_2_) and is absolutely important in hydrogen economy. In a water electrolyzer, water electrolysis is separated into two half-reactions: hydrogen evolution reaction (HER) happens at the cathode, i.e., 2H^+^ + 2e^−^ → H_2_ (under acidic conditions), while oxygen evolution reaction (OER) occurs at the anode, i.e., 2H_2_O → O_2_ + 4H^+^ + 4e^−^. Notably, OER is more kinetically sluggish compared to HER, as it is a four-electron transfer reaction, while HER requires only two electrons. To date, both of these half-reactions have relied on noble-metal-based electrocatalysts to reach their best performance, namely Pt for HER, and IrO_2_ and RuO_2_ for OER [1,2,3,4]. Despite their high activity, noble-metal-based electrocatalysts are expensive, and this limits their employment in real applications. In this context, progress has been made towards the exploration of non-precious-based metal electrocatalysts, while recent trends want transition metal-based and carbonaceous electrocatalysts to reach low overpotentials for HER and OER [5,6,7,8,9,10,11,12,13]. Moreover, due to the different mechanisms that govern HER and OER, it is quite often a good electrocatalyst that can perform at lower overpotential for HER to show poor activity for OER and vice versa. Therefore, it still remains quite a challenge to effectively employ highly active, stable, abundant and low-cost bifunctional electrocatalysts for water electrolysis that compete the performance of the precious metals ones [1,14].

Carbon nanohorns (CNHs) consist of single-graphene tubules, with highly strained conical ends that aggregate in larger spherical superstructures [15,16,17]. Their unique shape, chemical defects and crystal edges make them interesting material as electrocatalyst [15,16]. Specifically, CNHs have mostly been employed as supports, and have shown encouraging results in crucial electrocatalytic reactions involved in fuel cells, assisting in reaching superior performances as well as improved durability and stability [15]. In addition, doping with heteroatoms has proven to be an efficient method for increasing the catalytic activity of graphene-related materials [18,19,20,21] and specifically CNHs [22,23,24,25]. Nevertheless, effective, easy and inexpensive ways of incorporating heteroatom dopants is still challenging, since doping methods mostly involve tedious and expensive preparation techniques [15,26,27]. However, CNHs are still not fully exploited as electrocatalysts, since there are only very few works dealing with CNHs in HER [28,29] and OER [30] and none as bifunctional electrocatalyst for both.

Considering all of the aforementioned points, we embarked on the preparation of sulfur-doped CNHs (S-CNHs) and the subsequent assessment of their electrocatalytic ability. Herein, S-CNHs were prepared by employing a simple and inexpensive one-pot solvothermal method, fully characterized using complementary spectroscopic, thermal and microscopy imaging means, and their bifunctional nature to electrocatalyze the HER and OER was screened. Notably, for the first time, we report data showing excellent electrocatalytic activity and durability of S-CNHs for both HER and OER.

## 2. Materials and Methods

### 2.1. Preparation of ox-CNHs

For the preparation of ox-CNHs, 30 mg of pristine CNHs were treated with H_2_O_2_ (50 mL) under light irradiation at 120 °C for 3 h. Then, the dispersion was filtrated through PTFE membrane filter (pore size 0.2 μm), and the solid residue was extensively washed with deionized water and methanol. This procedure was repeated to obtain the ox-CNHs material as black powder.

### 2.2. Preparation of S-Doped CNHs

An amount of 50 mg of ox-CNHs and 250 mg of Lawesson’s reagent (St. Louis, MO, USA) were dispersed in 210 mL of diethylene glycol methyl ether and sonicated for 30 min. Afterwards, the mixture was placed into a 300 mL Teflon-lined stainless-steel autoclave reactor. The autoclave reactor was kept at 230 °C for 48 h. The dispersion was filtered over a PTFE filter (pore size 0.2 μm) and washed extensively with methanol. The solid residue was collected to obtain the S-doped CNH material.

### 2.3. General

Chemicals, reagents, and solvents were purchased from Sigma-Aldrich and used without further purification. Infrared (IR) spectra were obtained on a Fourier Transform IR spectrometer (Equinox 55 from Bruker Optics, Billerica, Massachusetts, US) equipped with a single reflection diamond attenuated total reflection (ATR) accessory (DuraSamp1IR II by SensIR Technologies, Danbury, CT, USA). Raman measurements were recorded with a Renishaw confocal spectrometer (Wotton-under-Edge, UK) at 514 nm. The data were obtained and analyzed with Renishaw Wire and Origin software (OriginPro 2018, Northampton, MA, USA). Thermogravimetric analysis was performed using a TGA Q500 V20.2 Build 27 instrument by TA (New Castle, DE, USA) in a nitrogen (purity > 99.999%) inert atmosphere. High-resolution TEM was performed employing an imaging-side aberration-corrected FEI Titan-Cube microscope working at 80 kV, equipped with a Cs corrector (CETCOR from CEOS GmbH, Heidelberg, Germany). XPS were recorded on a Kratos Axis Supra spectrometer, (Manchester, UK) equipped with a monochromated Al Kα X-ray source using an analyzer pass energy of 160 eV for survey spectra and 20 eV for the core level spectra. Spectra were recorded by setting the instrument to the hybrid lens mode and the slot mode providing approximately a 700 *×* 300 µm^2^ analysis area using charge neutralization. Regions were calibrated using the reference value BE(C 1s sp2) = 284.5 eV. All XPS spectra were analyzed using CASA XPS software (2.3.23, Teignmouth, Devon, UK). The XPS peaks were fitted to GL(70) Voigt lineshape (a combination of 70% Gaussian and 30% Lorentzian character), after performing a Shirley background subtraction. For the light-assisted oxidation reaction of CNHs the light source used was a 500 W halogen lamp, which was positioned 20 cm away from the reactor. Autoclave synthesis was undertaken in a Parr autoclave reactor with a 300 mL Teflon-lined stainless-steel vessel. All electrochemical measurements were carried out using an Metrohm Autolab PGSTAT128N potentiostat/galvanostat and were carried out at room temperature in a standard three-compartment electrochemical cell by using a platinum wire as a counter-electrode, an RDE with glassy carbon disk (geometric surface area: 0.0196 cm^2^) as a working electrode, and Hg/HgSO_4_ (0.5 M K_2_SO_4_) and Hg/HgO (0.1 M KOH) (AMEL S.r.l., Milan, Italy) as reference electrodes for HER and OER, respectively. LSV measurements for HER were carried out at room temperature in N_2_-saturated 0.5 M H_2_SO_4_. LSV measurements for OER were carried out at room temperature in O_2_-saturated 1.0 M KOH. The catalyst ink was prepared by dispersing 4.0 mg of the catalytic powder in a 1 mL mixture of water, isopropanol, and 5% Nafion (*v*/*v*/*v* = 4:1:0.02) and sonicated for 30 min prior to use. Before casting the electrocatalytic ink on the electrode’s surface, the working electrode was polished with 6-, 3- and 1-mm diamond pastes, rinsed with deionized water, and sonicated in double-distilled water. Afterwards, 8.5 μL aliquots of the electrocatalyst were casted on the electrode surface and were left to dry at room temperature. Electrochemical Impedance Spectroscopy (EIS) measurements (Metrohm Autolab, Utrecht, The Netherlands)) were conducted from 10^5^ to 10*^−^*^1^ Hz with an AC amplitude of 0.01 V, at a potential where significant HER and OER current was recorded, at −2 mA/cm^2^ for HER and at 3 mA/cm^2^ for OER. EIS data were fitted to Randles circuit. In EIS fit and simulation Chi square value is <0.001. The data were obtained and analyzed with Nova 2.1.4 software (Metrohm Autolab, Utrecht, The Netherlands).

## 3. Results and Discussion

**Figure 1:**
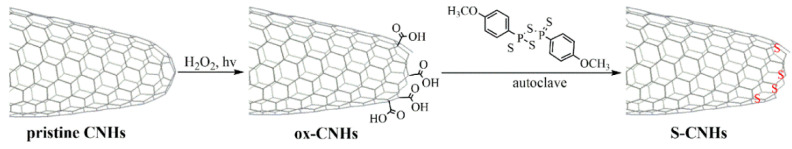
Illustrative preparation of S-CNHs.

**Figure 2:**
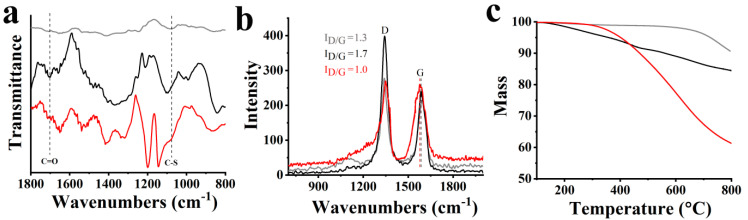
(**a**) ATR-IR spectra, (**b**) Raman spectra (514 nm), and (**c**) thermogravimetric analysis graphs obtained under nitrogen atmosphere of S-CNHs (red), ox-CNHs (black) and pristine CNHs (grey).

The preparation of S-doped CNHs was accomplished by treatment of oxidized CNHs (ox-CNHs) with Lawesson’s reagent, which was used as sulfur source, in the autoclave reactor, as shown in Figure 1. The doping of S atoms occurs mostly at the conical tips of ox-CNHs, where carbonyl groups are abundantly present. The doping mechanism can be briefly described as follows: initially, thioketone groups are formed, and afterwards, S atoms replace C atoms in the graphitic network [20,21]. ATR-IR and Raman spectra give proof of the successful realization of S-doped CNHs. Specifically, while the IR spectrum of ox-CNHs is governed by the characteristic carbonyl-related band at 1705 cm^−1^ due to stretching vibrations of the carboxylic acid moieties, the corresponding IR spectrum due to S-CNHs shows the decrease of the carbonyl band and the evolution of a new broad band at 1080 cm^−1^ related to C-S vibrations (Figure 2a). On the other hand, the Raman spectrum of pristine CNHs exhibits the characteristic D-band at 1341 cm^−1^, associated with defects, and the G-band at 1591 cm^−1^, related to the sp^2^ graphitic network. In the Raman spectrum of ox-CNHs, an increase of the intensity ratio I_D/G_ compared to pristine CNHs from 1.3 to 1.7, due to the change of hybridization from sp^2^ to sp^3^ upon oxidation, is evident. Concerning S-CNHs, there is a decrease of the I_D/G_ to 1.0 (Figure 2b) due to S-doping and partial restoration of the graphitic network [31]. Additionally, there is a shift of the G-band to lower frequencies by 8 cm^−1^, which is indicative of n-doping in S-CNHs [20].

Figure 2c shows the thermographs of pristine CNHs, ox-CNHs and S-CNHs that were recorded under nitrogen atmosphere. Pristine CNHs present an almost stable thermal profile up to 800 °C, while ox-CNHs show a 9% mass loss up to 500 °C due to the thermal decomposition of the carboxylic groups. Conversely, this is not shown in the thermograph of S-CNHs, where the thermal mass loss starts after 330 °C. The mass loss at higher temperatures is attributed to the thermal decomposition of the graphitic framework at sp^3^ defects close to where sulfur doping takes place.

**Figure 3:**
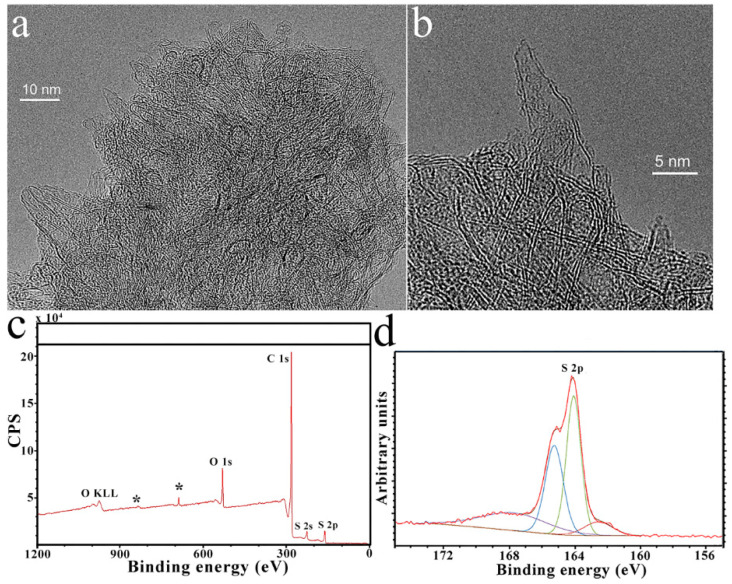
(**a**,**b**) HR-TEM micrographs showing the structure of S-CNHs. (**c**) XPS survey spectrum recorded on one of these S-CNHs samples, where S_2p_, C_1s_ and O_1s_ are observed. Impurities <1% due to F are shown with *. (**d**) High-resolution S_2p_ XPS of S-CNHs. The peaks are fitted to four components centered at 162.2, 164.1, 165.3 and 168.4, corresponding to S_p3/2_, S_p3/2_, S_p1/2_, and S-O, respectively.

HR-TEM imaging and XPS were employed to determine the morphology and the chemical composition and elemental content of S-CNHs. In more detail, HR-TEM micrographs (Figure 3a,b) show the characteristic morphology of those nanostructures and their aggregates’ organization. In the XPS survey spectrum of S-CNHs, characteristic peaks due to S_2p_, C_1s_, O_1s_ located at binding energies of ca. 164, 285 and 533, respectively, are evident (Figure 3c). Moreover, the S content was determined to be 3%, higher than that reported for S-doped graphene [31] and previously prepared S-doped CNHs [26]. To probe the chemical state of sulfur in S-CNHs high-resolution S_2p_ peaks were analyzed (Figure 3d). The S_2p_ peaks can be resolved into four peaks at binding energies of 162.2 (8.4%), 164.1 (70.6%), 165.3 (11.3%) and 168.4 (9.8%) eV, respectively. While the second and third deconvoluted peaks are related to sulfur bound as heteroatoms to aromatics, such as thiophene [31,32], the peaks at 162.2 and 168.4 eV arise from thiol and sulfone components [31,32]. The high percentage of the peaks corresponding to C-S related bonds confirms that doping of sulfur atoms happens mostly at the conical tips of CNHs where five-membered rings are located [16].

**Figure 4:**
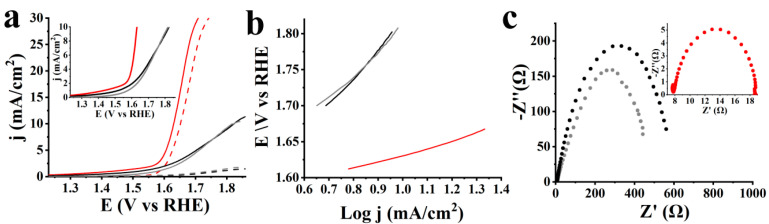
(**a**) LSVs, before (solid lines) and after 2000 cycles (dashed lines) for OER, (**b**) Tafel slopes, and (**c**) Nyquist plots of S-CNHs (red), ox-CNHs (black) and pristine CNHs (grey). Inset (**a**) LSVs at 10 mA/cm^2^; (**c**) Nyquist plot for S-CNHs. The LSVs obtained at 1600 rpm rotation speed and 5 mV/s scan rate.

Next, evaluating the electrocatalytic properties of S-CNHs against OER, linear sweep voltammetry (LSV) measurements were performed in O_2_ saturated 1.0 M aqueous KOH. A very important factor for evaluating the performance of OER electrocatalysts is the onset potential. However, since it is hard to determine the exact value, the potential at 10 mA/cm^2^ is commonly used as a benchmark. In OER, overpotential is the potential difference between the potential registered at a specific current density and the equilibrium potential, i.e., 1.23 V [1,2]. Figure 4a screens the polarization curves of S-CNHs, ox-CNHs and pristine CNHs. It is clear from the LSV curves and the recorded values of overpotentials registered at 10 mA/cm^2^ current density (Table 1) that S-CNHs possess the best electrocatalytic activity with the lowest potential at 1.63 V vs. RHE. Such a low overpotential is considered excellent for the OER, and is close to the overpotentials registered for noble metals [1,2,12]. On the contrary, the corresponding potential for both ox-CNHs and pristine CNHs was registered at 1.82 vs. RHE, 220 mV higher than that of S-CNHs. At the same time, S-CNHs reached high current density values compared to the other two reference materials, which is a prerequisite for an effective OER electrocatalyst. The superior electrocatalytic activity of S-CNHs is attributed to the presence of sulfur dopants, which induce a non-uniform spin density distribution due to the mismatch of the outermost orbitals of sulfur and carbon atoms, while further altering the charge density at nearby sites.

To define how swiftly the current increases with the applied overpotential, Tafel slopes were extracted from the LSV curves. Specifically, S-CNHs showed a low Tafel slope value of 80 mV/dec, as shown in Figure 4b. Low Tafel slope values are required for ideal electrocatalysts as they result in an improved OER rate under a constant increase of overpotential, which can be beneficial in real applications. The two reference materials, ox-CNHs and pristine CNHs, accordingly showed much higher Tafel slope values of 202 and 333 mV/dec, which correspond to slower OER kinetics. Electrochemical impedance spectroscopy (EIS) assays were performed to evaluate the OER kinetics. S-CNHs showed the smallest frequency semicircle in the Nyquist plot, which corresponds to a small charge transfer resistance (R_ct_) value of 10.8 Ω (Figure 4c). On the contrary, the very high R_ct_ values of the ox-CNHs and pristine CNHs, 560.5 and 450.5 Ω, respectively, reveal high charge transfer resistance. The higher R_ct_ value of ox-CNHs compared to pristine CNHs is justified by the disruption of the graphitic network due to the introduction of carboxylic species. Conversely, the low charge transfer resistance that was reported for S-CNHs arises from the increased conductivity due to the improvement of the graphitic degree resulting from the sulfur doping.

Stability is a crucial factor for the evaluation of the overall performance of an electrocatalyst. Figure 4a shows the LSV polarization curves for OER of S-CNHs as compared to that for ox-CNHs and pristine CNHs, after continuous cycling for 2000 cycles. Notably, negligible loss of the anodic current is noted for S-CNHs. As a striking difference, the two reference materials exhibit extremely huge losses of the anodic current that are unable to reach current density values at 10 mA/cm^2^. Electrochemical OER data for S-CNHs, ox-CNHs and pristine CNHs before and after 2000 cycles are summarized in Table 1.

**Figure 5:**
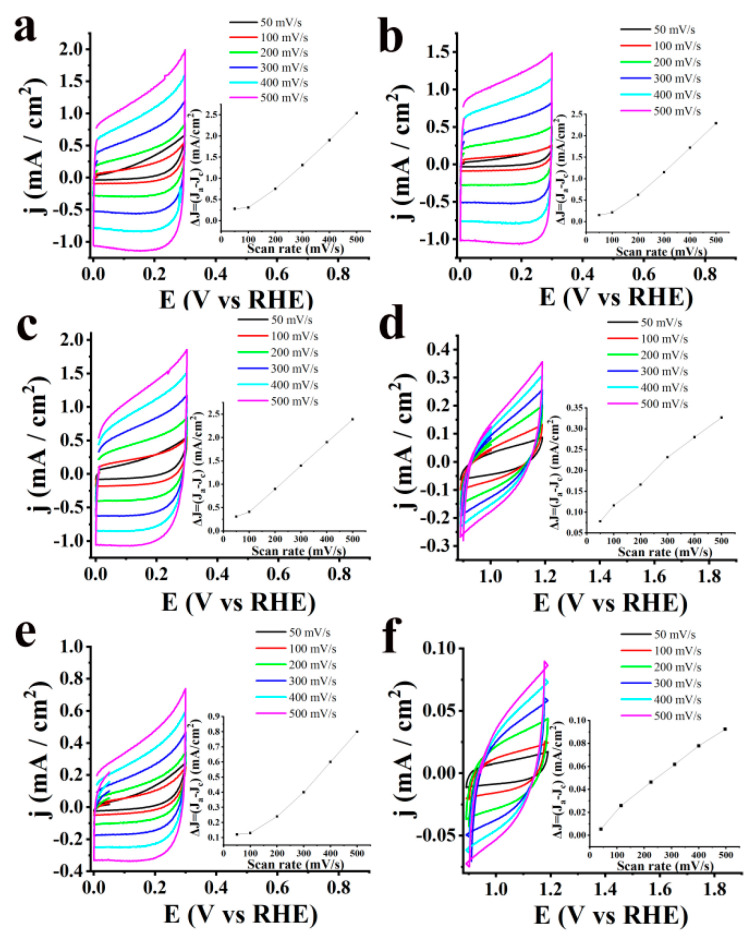
Cyclic voltamographs for OER of S-CNHs (**a**) before and (**b**) after 2000 cycles, ox-CNHs (**c**) before and (**d**) after 2000 cycles and pristine CNHs (**e**) before and (**f**) after 2000 cycles measured in a nitrogen saturated aqueous 1.0 M KOH, at a rotation speed of 1600 rpm and scan rates from 50 to 500 mV/s. Inset: Scan rate dependence of the current densities for the corresponding materials.

To get a better understanding of the charge transport flow in S-CNHs in OER, the electrochemically active surface area (ECSA) was estimated. In more detail, ECSA values were calculated based on the following equation [20,33,34,35,36]: ECSA = *C_dl_*/*C_s_*, where *C_dl_* stands for the electrochemical double-layer capacitance, while *C_s_* is the specific capacitance of a flat surface with 1 cm^2^ of real surface area with a value assumed to be 40 μF/cm^2^ for the flat electrode. For the estimation of the ECSA, cyclic voltamographs were recorded in a non-Faradaic region at scan rates of 50, 100, 200, 300, 400 and 500 mV/sec for all tested materials before and after 2000 cycles (Figure 5). ECSA values were obtained from *C_dl_* by plotting the *Δj = (j_a_ − j_c_)* at a given potential versus the scan rate as stated in the equation *C_dl_* = *d*(Δ*j*)/2*dV_b_*. Specifically, S-CNHs showed higher ECSA value of ~67.5 cm^2^. Consistent with the LSV polarization curves for OER, ox-CNHs and pristine CNHs present significantly lower ECSA, ca. ~60 and ~19.4 cm^2^, respectively. These values are in accordance with the electrocatalytic results towards OER, since the efficient accessibility of the active sites is related to higher ECSA values. Moreover, after cycling continuously for 2000 cycles, a slight decrease of the ECSA was noted for S-CNHs, ~61.25 cm^2^, while ox-CNHs and pristine CNHs exhibited huge drops at ~13.75 and ~4.7 cm^2^, respectively.

**Figure 6:**
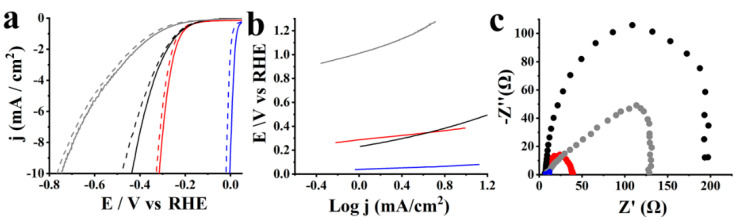
(**a**) LSVs, before (solid lines) and after 10,000 cycles (dashed lines) for HER, (**b**) Tafel slopes, and (**c**) Nyquist plots of S-CNHs (red), ox-CNHs (black), pristine CNHs (grey) and Pt/C (blue). The LSV polarization curves obtained at 1600 rpm rotation speed and 5 mV/s scan rate.

Next, the electrochemical properties of S-CNHs against HER were evaluated by performing LSV measurements in N_2_-saturated 0.5 M aqueous H_2_SO_4_. The polarization curves of S-CNHs, along with ox-CNHs and pristine CNHs as well as commercially available 20 wt% Pt on carbon black (Pt/C) as reference materials, can be seen in Figure 6a. The lowest onset potential is registered at −0.2 V vs. RHE for S-CNHs, which is more positive by 50 and 120 mV in comparison with the values noted for ox-CNHs (i.e., at −0.25 V) and pristine CNHs (i.e., at −0.32 V), respectively. In addition, potential values for HER are often compared at the functional current density for electrochemical water splitting of −10 mA/cm^2^, where sufficient hydrogen production is achieved, while faulty interpretations from intrinsic electroactivity are prevented. It is clear that S-CNHs show higher electrocatalytic activity at −0.31 V vs. RHE, shifted by 120 and 440 mV to more positive potentials compared to the value recorded for ox-CNHs (i.e., at −0.43 V) and pristine CNHs (i.e., at −0.75 V), respectively.

Furthermore, to define the rate-limiting step of HER, Tafel slopes were extracted from the LSV curves (Figure 6b). Additional analysis reveals that S-CNHs present a Tafel slope of 82 mV/dec, which is slightly higher than the one of Pt/C (i.e., 35 mV/dec). The latter demonstrates that the electrochemical desorption of adsorbed hydrogen atoms onto the electrode (Heyrovsky desorption) is the rate-limiting step for HER. On the other hand, HER of ox-CNHs and pristine CNHs is rate-limited by the initial adsorption of a proton onto electrode’s surface (Volmer adsorption), which is screened by the much higher Tafel slope values of 240 and 320 mV/dec, respectively. The easier kinetics of HER, revealed from the much lower Tafel slope for S-CNHs, signifies more reactive/accessible protons and water molecules that reach the electrochemical active sites [33].

Next, EIS assays were performed to evaluate the HER kinetics. EIS measurements further confirm the superior HER kinetics of S-CNHs (Figure 6c), which show the smallest R_ct_ value of 30.4 Ω, comparable to that of Pt/C (i.e., 6.1 Ω). On the other hand, ox-CNHs and pristine CNHs exhibited much higher R_ct_ values, 197 and 123.4 Ω, respectively. This tendency is in line with the results of Tafel slope values, which reveal improved kinetics and higher electrocatalyst activity of S-CNHs.

**Figure 7:**
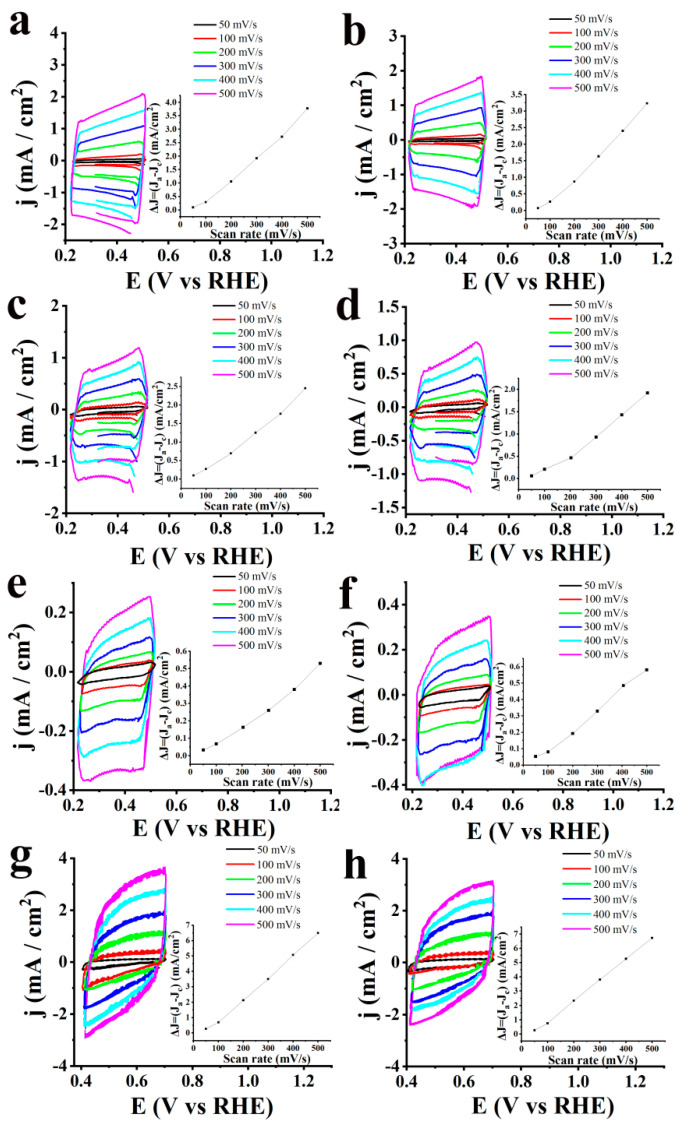
Cyclic voltamographs for HER of S-CNHs (**a**) before, and (**b**) after 10,000 cycles, ox-CNHs (**c**) before, and (**d**) after 10,000 cycles, pristine CNHs (**e**) before, and (**f**) after 10,000 cycles, and Pt/C (**g**) before, and (**h**) after 10,000 cycles, measured in a nitrogen saturated aqueous 0.5 M H_2_SO_4_ electrolyte, at a rotation speed of 1600 rpm and scan rates from 50 to 500 mV/s. Inset: Scan rate dependence of the current densities for the corresponding materials.

Additionally, the long-term stability of S-CNHs was assessed on the basis of durability studies. Figure 6a shows the LSV polarization curves for HER of S-CNHs in comparison to that for ox-CNHs, pristine CNHs and Pt/C, after ongoing cycling for 10,000 cycles, where negligible loss of the cathodic current is noted. Table 2 summarizes the HER-derived data for all tested materials, before and after 10,000 cycles. Finally, the ECSA values were also calculated. Cyclic voltamographs for HER of S-CNHs, as compared to that for ox-CNHs, pristine CNHs and Pt/C, in a non-Faradaic region were measured at scan rates of 50, 100, 200, 300, 400 and 500 mV/sec (Figure 7). Particularly, S-CNHs exhibit the higher ECSA value, ~205 cm^2^, while the equivalent values that are noted for ox-CNHs and pristine CNHs are significantly lower, i.e., ~130 and ~31.25 cm^2^, respectively. Similarly, the estimated ECSA values for the same materials after 10,000 cycles are remarkably lower than that recorded for S-CNHs.

## 4. Conclusions

In summary, we used a facile doping strategy to introduce sulfur atoms into the graphitic network of CNHs, and we tested S-CNHs against OER and HER. The S-CNHs proved to be a highly efficient bifunctional electrocatalyst towards water splitting due to the high S doping levels. Doping with sulfur effectively modified the electronic structure of CNHs, which along with high conductivity helped to achieve low overpotential values for both OER and HER. Specifically, S-CNHs showed low overpotential for oxygen and hydrogen evolution reaction, at 1.63 and −0.2 V vs. RHE, respectively, and excellent durability and stability. Additionally, S-CNHs exhibited significantly lower Tafel slope and lower current resistance compared to oxidized and pristine CNHs for both electrocatalytic reactions. The easy preparation method, high activity and excellent durability of S-CNHs make them a promising alternative for replacing noble-metal-based electrocatalysts for water splitting.

## Figures and Tables

**Table 1:** Electrochemical parameters for OER.

Catalyst	Potential (V vs. RHE) at 10 mA/cm^2^	Overpotential (mV vs. RHE) at 10 mA/cm^2^	Tafel Slope (mV/dec)	ECSA (cm^2^) ^b^	R_ct_ (Ω)
S-CNHs	1.63	400	80	67.5	10.8
S-CNHs ^a^	1.65	420	84	61.25	-
ox-CNHs	1.82	590	202	60	560.5
ox-CNHs ^a^	2.5	-	279	13.75	-
pristine CNHs	1.82	590	333	19.4	450.5
pristine CNHs ^a^	2.5	-	310	4.7	-

^a^ After 2000 cycles. ^b^ ±2% error.

**Table 2:** Electrochemical parameters for HER.

Catalyst	Onset Potential (V vs. RHE)	Potential (V vs. RHE) at −10 mA/cm^2^	Tafel Slope (mV/dec)	ECSA (cm^2^) ^b^	R_ct_ (Ω)
S-CNHs	−0.2	−0.29	82	205	30.4
S-CNHs ^a^	−0.21	−0.33	82	190	-
ox-CNHs	−0.25	−0.43	240	130	197
ox-CNHs ^a^	−0.26	−0.48	245	102	-
pristine CNHs	−0.34	−0.75	320	47.5	123.4
pristine CNHs ^a^	−0.35	−0.79	320	31.25	-
Pt/C	0.034	−0.0025	35	364	6.1
Pt/C ^a^	0.011	−0.02	35	350	-

^a^ After 10,000 cycles. ^b^ ±2% error.
